# Maternal Dietary Diversity and Small for Gestational Age: The Effect Modification by Pre-Pregnancy Body Mass Index and Gestational Weight Gain in a Prospective Study within Rural Sichuan, China (2021–2022)

**DOI:** 10.3390/nu15173669

**Published:** 2023-08-22

**Authors:** Chang Sun, Yuju Wu, Zhengjie Cai, Linhua Li, Jieyuan Feng, Amy van Grieken, Hein Raat, Scott Rozelle, Huan Zhou

**Affiliations:** 1 West China School of Public Health and West China Fourth Hospital, Sichuan University, Chengdu 610041, China; changsun233@163.com (C.S.);; 2 Rural Education Action Program, Freeman Spogli Institute for International Studies, Stanford University, Palo Alto, CA 94305, USA; 3 Department of Public Health, Erasmus MC, University Medical Center Rotterdam, 3015 GD Rotterdam, The Netherlands

**Keywords:** maternal dietary diversity, small for gestational age, underweight prior to pregnancy, prospective study, synergistic effects

## Abstract

Infants born small for gestational age (SGA) remains a significant global public health concern, with potential interconnections among maternal diet, pre-pregnancy BMI, gestational weight gain (GWG), and SGA. This prospective study investigated the association between dietary diversity (DD) during pregnancy and the risk of SGA, as well as the synergistic effect of DD with pre-pregnancy BMI and GWG on SGA. Maternal dietary intake during pregnancy was assessed using 24 h dietary recalls, and dietary diversity scores (DDS) were calculated based on the FAO’s Minimum Dietary Diversity for Women index. Infant information was followed up. The Poisson regression model was employed to determine the association between maternal DD and SGA. Interactions between DD and pre-pregnancy BMI or GWG were evaluated under additive and multiplicative models. Among the 560 singleton live births, 62 (11.07%) were classified as SGA. After adjusting for potential confounders, the DDS exhibited a protective effect against SGA (aRR: 0.76; 95% CI: 0.62–0.95). DD modified the association between being underweight prior to pregnancy and SGA on the additive scale (interaction contrast ratio = 7.39; 95% CI: 5.84, 8.94). These findings suggest that improving dietary diversity during pregnancy, particularly among women with a low pre-pregnancy BMI, may be a feasible strategy to reduce the risk of SGA newborns.

## 1. Introduction

Ensuring sufficient nutritional status during pregnancy is crucial for promoting optimal fetal development and growth. The extensive literature has consistently indicated significant associations between prenatal maternal nutritional factors and pregnancy outcomes, particularly via intrauterine growth [1]. Abnormalities in intrauterine growth patterns can lead to atypical birth weight, such as the occurrence of a small for gestational age (SGA) infant. SGA is a condition in which an infant is born at a weight below the 10th percentile of the population-specific birth weight for a given region, taking into account the infant’s sex and weeks of gestation [2]. Accumulated evidence suggests that SGA not only increases the risk of perinatal morbidity and mortality [3,4] but also carries potential long-term consequences, such as delayed development in childhood [5], poor school performance in adolescence [6], and metabolic disease in adulthood [7].

In low-and-middle-income countries (LMICs), where an estimated 250 million children under five years of age are at an elevated risk of developmental delay, the prevalence of SGA has been found to range from 7% to higher than 50% [3]. Additionally, approximately 21% of neonatal deaths in LMICs can be attributed to SGA [8]. Despite the magnitude of the problem, few studies have examined modifiable risk factors and causes of SGA in LMIC settings.

While there are multiple risk factors for impaired fetal growth, maternal nutrition during pregnancy has been identified as one of the factors that can be addressed [9]. Specifically, pre-pregnancy nutritional status, measured through body mass index (BMI) and gestational weight gain (GWG), both of which are largely determined by nutritional intake during pregnancy, have been identified in the literature as strong predictors of maternal and infant outcomes [10]. According to results from multiple systematic reviews and meta-analyses, being underweight prior to pregnancy (OR, 1.85; 95% CI, 1.69–2.02) or having low weight gain during pregnancy (USA/Europe (OR 1.51; CI 1.39, 1.63); Asia (1.63; 1.45, 1.82)) were both associated with a higher risk of giving birth to an SGA infant [11,12]. As well, insufficient maternal nutrient intake during gestation restricts the availability of essential nutrients required for optimal fetal development [7]. Consequently, the quality of the maternal diet has been suspected to play an important role in determining infant outcomes.

Because pregnant women living in low-income settings have a higher risk of developing nutrient deficiencies [13], dietary diversity (DD), or the consumption of food items from ten food groups within a specific timeframe, has been a widely used metric for measuring the micronutrient adequacy of a woman’s diet. Evidence demonstrates that the utilization of DD to develop dietary recommendations can be valuable in reducing the connections between adverse birth outcomes and maternal undernutrition, especially for vulnerable populations [14]. According to studies conducted in Tanzania and Spain, women with higher DD presented a lower risk of SGA [14,15]. A longitudinal study in Sri Lanka revealed that carbohydrate intake during pregnancy as well as pre-pregnancy BMI both had significant impacts on maternal weight gain during pregnancy and infant birth weight outcomes [10]. These findings suggest that there are overlapping associations between maternal diet, pre-pregnancy BMI, GWG, and birth weight outcomes. Notably, however, no longitudinal study has investigated the relationship between maternal DD, pre-pregnancy BMI, GWG, and SGA.

A study analyzing birth cohorts from 14 LMICs revealed that China has the fifth highest prevalence of SGA births in the world [16]. Despite China’s rapid economic growth over the past few decades, rural communities in western China still exhibit characteristics of an underdeveloped economy with lower population health literacy that make these areas relatively similar to the underdeveloped settings observed in other LMICs [17]. Importantly, there is a notable absence of research focused on SGA in this specific region.

To address the above gap in the literature, our study aims to prospectively investigate the relationship between pre-pregnancy BMI, GWG, DD, and the incidence of SGA among pregnant women residing in the rural communities of western China. We aim to provide empirical evidence regarding modifiable risk factors associated with SGA in understudied populations within LMICs. To meet this overall goal, this paper’s specific objectives are to (a) determine the incidence of neonatal SGA in the study population using China’s national standards; (b) examine whether lower maternal DD after conception is associated with an increased risk of SGA; and (c) explore the potential synergistic effect of DD with two other nutrition-related exposures, pre-pregnancy BMI and GWG, on the occurrence of SGA.

## 2. Materials and Methods

### 2.1. Study Design and Participants

This study uses data from a longitudinal cohort study of pregnant women conducted in rural counties within Nanchong Prefecture of Sichuan Province, China. Nanchong represents a relatively economically disadvantaged prefecture in Southwestern China, with seven out of the nine counties within the prefecture holding national or provincial designations as poverty-stricken counties. To select the study sample, a multistage cluster sampling protocol was followed. First, four out of the seven nationally designated poverty counties were randomly selected, and all townships located in these four counties were included in the sampling frame. Next, all urban townships and townships with less than 10,000 residents were excluded. Stratified by four counties, 80 townships were selected randomly in total. Baseline data were collected between July and August of 2021, and follow-up data were collected between May and June 2022 after all participants had delivered. Finally, all participants provided their informed consent prior to enrollment and understood that their participation was purely voluntary. This study was approved by the Sichuan University Medical Ethics Committee (K2019046) (Chengdu, China), Stanford University (44312) (Stanford, CA, USA), and the University of Nevada, Reno (1737966-1) (Reno, NV, USA).

Sample participants were selected based on a list of all pregnant women in the selected townships provided by the local perinatal healthcare system. First, all pregnant women who were at least 14 weeks into gestation were enrolled in the study. If a participant had either a multifetal gestation or a fetus suspected to have anomalies, they were excluded from the study. Second, trained enumerators conducted computer-assisted home visit interviews to collect information from all participants using a structured and pre-tested questionnaire. Third, trained nurses obtained the height, weight, and hemoglobin level of all participants. The resulting baseline sample comprised 629 pregnant women participants. During follow-up, 9 participants were recorded to have a pregnancy loss, 54 participants moved or were lost to follow-up, and 6 participants had missing information on key variables or had reported a difference between dietary intake on the previous day and their usual dietary habits. As a result, a total of 560 mother–newborn pairs were included in the final analysis.

### 2.2. Exposure Assessment

#### 2.2.1. Dietary Diversity during Pregnancy

In this study, we used a participant’s dietary diversity score (DDS) as a study exposure to examine the dose–response relationship with SGA outcomes. The dichotomous minimum dietary diversity (MDD) was evaluated and utilized in further analysis. At baseline, we accessed dietary diversity during pregnancy with a questionnaire asking participants to recall all food items they had consumed in the 24 h prior to survey conduction. All food recalls were administered using the Multiple-Pass Method, which has been found to reduce error in dietary measurement by aiding an interviewee to recall in detail what they consumed the previous day [18]. Based off a participant’s answers to the recall questionnaire, we computed a DDS for each participant. In this study, we calculated the DDS and MDD according to guidelines developed by the Food and Agriculture Organization (FAO, Rome, Italy) regarding the Minimum Dietary Diversity for Women (MDD-W) [19]. The MDD-W was proposed for adoption in LMIC regions as a population-level proxy measure of micronutrient adequacy for women of reproductive age. Research has demonstrated a direct association between the MDD-W and the mean nutrient adequacy of 11 vital micronutrients [19].

To calculate each participant’s DDS, we classified every food item a participant reported consuming into 1 of 10 food groups: (1) starchy staples; (2) beans and peas; (3) nuts and seeds; (4) dairy; (5) flesh foods (meat, fish); (6) eggs; (7) vitamin A-rich dark green vegetables; (8) other vitamin A-rich fruits and vegetables; (9) other vegetables; and (10) other fruits. For food items with a variety of ingredients, such as complex dishes, we categorized it according to the main component. If a participant reported consuming at least one food item in a food group, consumption for that food group was recorded as ‘yes;’ otherwise, it was recorded as ‘no.’ To arrive at a participant’s final DDS, each food group marked as consumed added 1 point to their DDS, with the maximum score being 10. The criterion for achieving MDD was consuming food items from at least 5 of the 10 food groups. Thus, we defined a DDS of five or higher as meeting the MDD.

#### 2.2.2. Maternal Pre-Pregnancy Body Mass Index (BMI, kg/m^2^)

At baseline, the height and weight of the participants was measured using a unified portable stadiometer and scales. All participants were asked to self-report their weight before pregnancy. The pre-pregnancy BMI was calculated by dividing a participant’s self-reported pre-pregnancy weight (in kilograms) by their height (in meters). In this study, maternal pre-pregnancy BMI was categorized into one of four groups: underweight (BMI < 18.5 kg/m^2^), normal weight (18.5 ≤ BMI < 24.0 kg/m^2^), overweight (24.0 ≤ BMI < 28.0 kg/m^2^), and obese (BMI ≥ 28.0 kg/m^2^) according to China’s national guidelines [20] instead of the WHO’s guideline (which would use cut-off values of 18.5 kg/m^2^, 25 kg/m^2^, and 30 kg/m^2^). This is because women of Asian descent tend to have a higher body fat mass, lower lean body mass, and higher cardiometabolic risks at similar body mass indices as women of European descent (BMI) [21].

#### 2.2.3. Gestational Weight Gain (GWG)

We calculated the GWG of the participants by subtracting their self-reported pre-pregnancy weight from their measured weight at baseline. To evaluate GWG, we used guidelines from the ‘Technical Specification for Weight Monitoring and Evaluation during Pregnancy in Chinese Women’ (Group Standard) (T/CNSS 009-2021). Studies from China have suggested that Chinese mothers should have a GWG lower than the international standard proposed by the Institute of Medicine (IOM) [22,23]. We calculated the lower limit of the GWG for a participant’s current week of pregnancy by taking into account the current stage of pregnancy (or the gestational age of the fetus) and referring to the recommended range of weekly pregnancy weight gain according to a participant’s pre-pregnancy BMI category. If a participant’s weight gain during their current week of pregnancy fell below the recommended lower limit, they were classified as having a GWG below the guidelines [24].

### 2.3. Outcome Measures

At follow-up, birth-related outcomes, including birth weight (with weighing accuracy within 1 g), infant sex, and gestational age (in weeks) at birth, were obtained from infants’ birth certificates. In this study, we defined SGA as a birth weight that falls below the tenth percentile for an infant’s gestational age, when compared to the expected weight for Chinese infants of the same gestational age and sex. This definition is based on the growth standards for newborns by gestational age specific to Chinese infants as specified by the Health Industry Standard of China (WS/T 800-2022) [25].

### 2.4. Covariates

Potential common confounders associated with SGA were identified through a comprehensive literature review and were collected at baseline or follow-up. To detect anemia, blood from the ring finger of participants was collected at baseline by trained professionals and their hemoglobin levels were measured using a HemoCue HB201+ system (Angelholm, Sweden, 2020). According to the WHO’s recommended diagnostic criteria for anemia in pregnant women, a hemoglobin value of less than 110 g/L indicates anemia [26]. Various confounders such as maternal age at delivery, maternal education (≤9, 10–12, 13–15, ≥16 years), parity (primipara/multipara), abortion history, previously birthing a low birth weight (LBW) baby (yes/no), annual per capita income of the family (in CNY), household assets (whether the household owned or had access to a water heater, washing machine, refrigerator, air conditioner, television, computer, motorcycle, and car or truck), gestational hypertension throughout pregnancy (yes/no), iron supplementation throughout pregnancy (yes/no), and folic acid supplementation throughout pregnancy (yes/no) were assessed in the follow-up survey. We calculated the socioeconomic status (SES) of participants by referring to data on maternal education, the annual per capita income of the family, and household assets. A principal component analysis (PCA) was performed to arrive at each participant’s SES score, which was then sorted into one of three categories: low level (Q1), medium level (Q2), and high level (Q3).

### 2.5. Statistical Analyses

To ensure the statistical power of our sample, we performed a post hoc power analysis utilizing a small effect size (f^2^ estimate of 0.02), an α value of 0.05, and a sample size of 560 for a conservative assessment of power. The analysis yielded a post hoc power of 0.86, indicating that the sample size had sufficient power to detect the intended effects.

To describe the participant characteristics and to test for univariate associations of participant characteristics with SGA, the maternal and infant characteristics of participants with or without infants born SGA were compared with Student’s t-tests and chi-square tests for continuous and categorical variables, respectively.

To examine the factors contributing to SGA, multivariate Log-Poisson regressions with robust standard errors were conducted to estimate the risk ratios and 95% confidence intervals for the association of SGA with pre-pregnancy BMI, DDS, GWG, and other covariates with significant univariate distribution differences. The significant variables were selected for further study.

To explore potential interactions between risk factors for SGA, we assessed effect measure modification (EMM) by inadequate MDD (DDS < 5). Specifically, we examined the impact of pre-pregnancy underweight BMI and inadequate GWG on SGA, considering both additive and multiplicative scales. This was performed to determine whether the joint effect of two exposures (inadequate MDD and another) had greater effects on the risk of SGA than would be expected from the independent effects of each exposure.

To analyze EMM on the additive scale, for each combination of variables, we generated four exposure categories. These variables were modeled using Log-Poisson regressions adjusted for maternal age, gestational hypertension, SES, inadequate GWG, or pre-pregnancy underweight BMI. The output from these models was then used to estimate three interaction statistics: interaction contrast ratio (ICR), attributable proportion (AP), and synergy index (S). ICR is the excess risk due to interaction relative to the risk without either exposure. AP is the proportion of disease attributable to interaction among individuals with both exposures. S is the ratio of the observed excess risk in individuals exposed to both factors relative to the expected excess risk assuming that both exposures are independent risk factors (i.e., under the assumption of no additive interaction). For these metrics, 95% CIs were estimated using the delta method [27]. An ICR and AP > 0 and an S > 1 signify a positive departure from additivity. To analyze EMM on the multiplicative scale, models with the interaction term were tested with multiplicative interaction indexes (MIIs) [28], where an MII > 1 signifies a greater than multiplicative interaction.

All statistical analyses were performed using STATA 16.0 with a two-sided significance level of *p* = 0.05.

## 3. Results

### 3.1. Summary Statistics

A total of 560 eligible mother–infant pairs were included in our final analysis. Table 1 presents the characteristics of the mothers and infants, along with the univariable comparison based on infant SGA status. In total, 62 (11.07%) of the infants were born SGA. Significant differences were observed between participants with and without infants born SGA in terms of maternal age, socioeconomic status (SES), DD (measured as continuous DDS and categorical MDD), pre-pregnancy BMI classification, and gestational hypertension status (*p* < 0.05).

### 3.2. Associations of Infant SGA with Maternal Risk Factors

Table 2 presents the findings of the multivariate Poisson regression model examining the associations of infant SGA with several potential maternal risk factors. We find that a participant’s DDS (RR = 0.76, 95% CI: 0.62–0.95) and age (RR = 0.93, 95% CI: 0.91–0.96) both had a protective effect on the occurrence of infant SGA. On the other hand, inadequate GWG was found to have a significant association with an elevated risk of birthing an SGA infant (RR = 2.34, 95% CI: 1.44–3.83). Having a pre-pregnancy BMI classified as underweight (compared to those with a normal pre-pregnancy BMI) was also associated with an increased risk of infant SGA (RR = 1.94, 95% CI: 1.62–2.32), as was having gestational hypertension (RR = 3.03, 95% CI: 1.25–7.32). Finally, no significant associations were found between either a participant’s SES or their intake of folic acid supplements during pregnancy and the likelihood of birthing an SGA infant.

### 3.3. Associations of Infant SGA with MDD, GWG, and Underweight Pre-Pregnancy BMI

Table 3 displays the associations between infant SGA and various combinations of meeting/not meeting the MDD with adequate/inadequate GWG, or with/without a pre-pregnancy BMI classified as underweight.

The findings indicate that participants with inadequate GWG and those that had either met the MDD (aRR = 2.21, 95% CI = 1.16–4.19) or had not met the MDD (aRR = 6.13, 95% CI =2.89–12.99) had a significantly higher risk of birthing an SGA infant compared to those with adequate GWG and had met the MDD.

Participants with a pre-pregnancy BMI classified as underweight and those that did not meet the MDD had an increased risk of having an SGA infant (aRR = 9.02, 95% CI = 7.71–10.55). We find, however, that participants with a pre-pregnancy BMI classified as underweight that had met the MDD did not have a significant risk of birthing an SGA infant, suggesting that meeting the MDD may partially mitigate the risk of SGA associated with having a pre-pregnancy underweight BMI.

Table 3 also shows a significant additive interaction between a pre-pregnancy underweight BMI and not meeting the MDD. Our analysis revealed a relative excess risk of 7.39 for the additive interaction of not meeting the MDD with a pre-pregnancy underweight BMI. Furthermore, we found that approximately 82% of the incidence of SGA in infants that had been exposed to both risk factors (maternal pre-pregnancy underweight BMI and not meeting MDD) was attributable to the additive interaction. Notably, the incidence of SGA in infants that had been exposed to both risk factors was found to be 12.78 times higher than the sum of incidences in infants exposed to a single risk factor.

Finally, regarding the effect modification of meeting the MDD on GWG, the attributable proportion (AP) was found to be statistically significant. Specifically, 54.4% of the infants that had been exposed to both inadequate GWG and their mothers had not met the MDD had an SGA incidence that could be attributed to the additive interaction. However, considering that the interaction contrast ratio (ICR) is the priority indicator for assessing additive interaction effects rather than the AP or S [29], this study could not determine the presence of an interaction effect between MDD and GWG at present. Furthermore, the multiplicative interaction between the two combinations was not statistically significant.

## 4. Discussion

In this study, we used data from a sample of pregnant women living in rural areas of western China to assess the association of maternal prenatal dietary diversity (DD) with the likelihood of giving birth to an SGA infant. This paper also explored whether maternal DD acted synergistically with two other nutrition-related factors, pre-pregnancy BMI and GWG. In reviewing the main results of this study, after adjusting for confounding factors, the research team found that participants with more diverse diets had lower likelihoods of delivering an SGA infant. The analysis also demonstrated that adequate MDD (or consuming food items from at least 5 of the 10 food categories) modified the association between having an underweight pre-pregnancy BMI and the incidence of delivering an SGA infant. Specifically, the research established that the combined effect of being underweight prior to pregnancy and having inadequate MDD increased a mother’s risk of delivering an SGA infant beyond what would be expected from the individual effects alone (that is, either only being underweight prior to pregnancy (and not having inadequate MDD), or only having inadequate MDD). The findings highlight maternal prenatal DD as a potentially modifiable factor for preventing adverse infant outcomes, particularly for pregnant women that were underweight before pregnancy.

MDD was specifically developed to assess the overall dietary quality and diversity of women of reproductive age living in LMICs [19]. Although several studies have examined the association between MDD and the risk of giving birth to SGA infants, the results in the literature are often inconsistent. For example, a prospective study conducted in urban regions of Tanzania reported no association between MDD and SGA [30]. In contrast, two other studies, including a cohort study in Tanzania [14] and a case-control study in Spain [15], reported significant effect estimates. The results of the latter two investigations are consistent with this study’s finding of a reduced risk ratio between maternal MDD and infant SGA.

According to our analysis, the research team believes that the association between maternal MDD and infant SGA may be explained by several factors. First, meeting the MDD has been validated to offer adequate amounts of key micronutrients essential for immune system functioning and tissue growth, including folate, calcium, iron, zinc, and key vitamins [19,31,32]. Having inadequate micronutrient intake has been associated with various adverse pregnancy outcomes, including SGA, by disrupting the balance between proinflammatory and anti-inflammatory processes that may lead to disruptions in the functioning of the placenta [33]. Second, a diet characterized by a high variety of micronutrients often leads to reduced consumption of energy-dense foods that offer little nutritional value [34,35]. Previous studies have demonstrated that pregnant women that meet the MDD are much more likely to consume at least one animal-source food and either legumes or nuts/seeds [36]. Third, Yang et al. observed a positive correlation between MDD and sufficient energy intake as well as improved diet quality in pregnant women [30]. Studies on protein–energy supplementation have indicated the role of energy and protein intake in preventing SGA infants [37].

The current study is also the first to report the synergistic effect of low pre-pregnancy BMI and low maternal DD on the risk of giving birth to an SGA infant. Numerous studies have found that being underweight prior to pregnancy is associated with a higher risk of giving birth to an SGA infant [11]. Previous research has shown an inverted U-shaped relationship between DD and BMI in general adult populations. Specifically, consuming a less diverse diet was found to be associated with a lower BMI in participants that were underweight [38]. The same study also found that a decrease in one standard deviation in DD was found to correspond to a 2.3% decrease in BMI. Our findings suggest that increasing DD during pregnancy may help reduce the occurrence of SGA infants among women that were underweight before pregnancy. The reason for this could be attributed to the fact that consuming a wider variety of foods helps address deficiencies in protein, energy, and essential micronutrients that are crucial for optimal fetal growth and development. Notably, findings from Juan et al. (2020) revealed that the risk of birthing an SGA infant in underweight women can be mitigated if they consume foods rich in proteins, vitamins, and omega-3 marine fatty acids [39]. The consumption of foods rich in proteins, vitamins, and fatty acids becomes particularly significant considering the results of a cross-sectional study conducted on pregnant women in Japan which found that underweight participants had lower intakes of protein, iron, and folic acid [40]. Emphasizing the importance of consuming a diverse diet is crucial for ensuring healthy infant outcomes among pregnant women who may be vulnerable to malnutrition.

Our study had several strengths. First, the research was based on a prospective cohort study that allowed us to assess dietary status in the middle-to-late stages of pregnancy and enabled longitudinal research on DD. Second, the analysis adjusted for important potential confounders, including BMI, GWG, and gestational hypertension, which are often not well-controlled for in other studies. Third, this research provides epidemiologic evidence for synergism between DD and SGA, emphasizing the significance of DD during pregnancy, especially in high-risk populations.

This study also had several limitations. Despite careful consideration of known risk factors and potential confounders, residual confounding factors cannot be completely ruled out. This study’s data on pre-pregnancy body weight were also self-reported by participants after their pregnancy, a fact that could have introduced the possibility of recall bias, although this bias has been generally estimated to be around 1 kg and considered acceptable [41]. Finally, the analysis was based on a single 24 h set of dietary data, which might introduce random within-person errors. Nevertheless, this is consistent with the FAO’s definition of MDD-W which focuses on population-level interpretations rather than day-to-day variability in individual intakes [19]. Research has also shown that using multiple or single 24 h dietary recalls does not materially change the assessment of the relationship between dietary diversity and birth outcomes [14]. In future research, there are opportunities to refine the indicator measurements. Moreover, exploring larger sample populations could facilitate the identification of subtle patterns and correlations that might be obscured within smaller cohorts.

## 5. Conclusions

Considering the potentially harmful risks on both mother and fetus associated with inappropriate supplement dosages and adverse reactions, as highlighted by previous studies [42,43], as well as the convenience of obtaining supplements, dietary diversity can be considered a safe and cost-effective public health and clinical intervention, particularly in the context where food sources are easily accessible and secure. Furthermore, dietary diversity can offer advantages that may not be provided by prenatal supplements [44], such as the improvement in maternal dietary habits which can have potential long-term health benefits. In summary, this study suggests that improving dietary diversity during pregnancy, particularly among women with a low pre-pregnancy BMI, may be a particularly feasible strategy to reduce the risk of newborn SGA.

## Figures and Tables

**Table 1:** Maternal and infant characteristics and their univariable comparisons by infant SGA status in a prospective study within rural Sichuan, China (2021–2022) (*N* = 560).

Characteristics	Total Sample	By Infant SGA Status		*p*-Value
	(*N* = 560)	Infant Not SGA (*n* = 498)	Infant Is SGA (*n* = 62)	
Infant characteristics				
Gender (male)	276 (49.29)	246 (49.39)	30 (48.39)	0.91
Gestational age (weeks)	38.74 ± 1.37	38.73 ± 1.30	38.78 ± 1.89	0.84
Gestational age < 37 weeks	26 (4.64)	20 (4.01)	6 (9.67)	0.04
Birth weight (g)	3274.77 ± 505.79	3350.06 ± 440.44	2556.11 ± 407.79	<0.001
Birth weight < 2500 g	27 (4.82)	8 (1.60)	19 (30.65)	<0.001
Length (cm)	49.66 ± 1.59	49.84 ± 1.36	48.35 ± 2.58	<0.001
Maternal characteristics				
Age (years)	28.17 ± 4.69	28.38 ± 4.61	26.53 ± 5.00	0.003
Primipara	82 (14.64)	69 (13.86)	13 (20.97)	0.14
SES				
Low	184 (32.86)	156 (31.33)	28 (45.16)	0.004
Medium	180 (32.14)	156 (31.33)	24 (38.71)	
High	196 (35.00)	186 (37.35)	10 (16.13)	
DDS	6.26 ± 1.49	6.37 ± 1.47	5.45 ± 1.40	<0.001
Inadequate MDD	73 (13.04)	55 (11.04)	18 (29.03)	<0.001
Inadequate GWG	260 (46.43)	215 (43.17)	45 (72.58)	<0.001
Pre-pregnancy BMI (kg/m^2^)	23.34 ± 3.72	23.33 ± 3.66	23.45 ± 4.19	0.80
Pre-pregnancy BMI				
Underweight	36 (6.43)	27 (5.42)	9 (14.52)	0.03 ^a^
Normal	312 (55.71)	283 (56.83)	29 (46.77)	
Overweight/obese	212 (37.86)	188 (37.75)	24 (38.71)	
Gestational hypertension	29 (5.18)	19 (3.82)	10 (16.13)	0.001 ^a^
Anemic	298 (53.21)	265 (53.21)	33 (53.23)	0.99
Number of miscarriages	0.94 ± 0.98	0.94 ± 0.99	0.98 ± 0.91	0.74
Had previous LBW infant	24 (4.29)	23 (4.62)	1 (1.61)	0.23 ^a^
Iron supplementation	385 (68.75)	344 (69.08)	41 (66.13)	0.64
Folic acid supplementation	518 (92.50)	464 (93.17)	54 (87.10)	0.09

SGA, small for gestational age; SES, socioeconomic status; DDS, dietary diversity score (a possible range of 0–10); MDD, minimum dietary diversity (DDS ≥ 5); BMI, body mass index; LBW, low birth weight. ^a^ tested by Fisher’s exact test. Other unmarked *p*-values were determined using Student’s *t*-tests for continuous variables and chi-square tests for categorical variables.

**Table 2:** Associations of infant SGA status with maternal risk factors from univariate and multivariate Poisson regression model in a prospective study within rural Sichuan, China (2021–2022) (*N* = 560).

Factors	Reference Group	Univariate RR (95% CI)	Multivariate RR (95% CI)	*p*-Value
DDS	-	0.71 (0.57, 0.89)	0.77 (0.61, 0.97)	0.03
Inadequate gestational weight gain	adequate	2.87 (1.64, 5.04)	2.22 (1.42, 3.37)	<0.001
Underweight pre-pregnancy BMI	normal	2.71 (1.69, 4.32)	2.01 (1.78, 2.27)	<0.001
Overweight pre-pregnancy BMI		1.27 (0.88, 1.83)	1.10 (0.77, 1.57)	0.60
Gestational hypertension	no gestational hypertension	3.35 (1.28, 8.78)	2.74 (1.05, 7.11)	0.03
Medium SES	low SES	0.91 (0.71, 1.17)	0.96(0.71, 1.3)	0.79
High SES		0.34 (0.13, 0.89)	0.45(0.18, 1.15)	0.09
Maternal age	-	0.93 (0.9, 0.96)	0.94 (0.91, 0.96)	<0.001
Took folic acid supplementation	did not take folic acid supplementation	0.55 (0.24, 1.3)	0.65 (0.34, 1.22)	0.18

SGA, small for gestational age; RR, risk ratio; DDS, dietary diversity score; BMI, body mass index; SES, socioeconomic status.

**Table 3:** Additive and multiplicative effect measure modification by MDD for the multivariable-adjusted associations between inadequate GWG/pre-pregnancy underweight and SGA in a prospective study within rural Sichuan, China (2021–2022) (*N* = 560).

Exposure Category		aRR (95% CI) ^a^	Additive Interaction Statistics			Multiplicative Interaction Statistics
			ICR (95% CI) (Null Hypothesis = 0)	AP (95% CI) (Null Hypothesis = 0)	S (95% CI) (Null Hypothesis = 1)	MII (95% CI) (Null Hypothesis = 1)
Inadequate GWG	Inadequate MDD		3.33 (−0.66, 7.32)	0.54 * (0.13, 0.95)	2.85 (0.74, 11.05)	2.08 (0.70, 6.12)
no	no	1 (ref.)				
yes	no	2.21 ** (1.16, 4.19)				
no	yes	1.59 (0.45, 5.61)				
yes	yes	6.13 ** (2.89, 12.99)				
Underweight pre-pregnancy BMI	Inadequate MDD		7.39 ** (5.84, 8.94)	0.82 * (0.72, 0.92)	12.79 ** (3.14, 52.03)	2.05 (0.69, 6.13)
no	no	1 (ref.)				
yes	no	0.85 (0.55, 1.32)				
no	yes	1.78 (0.92, 3.43)				
yes	yes	9.02 ** (7.71, 10.55)				

MDD, minimum dietary diversity; GWG, gestational weight gain; SGA, small for gestational age; aRR, adjusted risk ratio; ICR, interaction contrast ratio; AP, attributable proportion; S, synergy index; MII, multiplicative interaction indexes. ^a^ Multivariate Poisson regression analysis was performed with adjustments for maternal age, gestational hypertension, SES, inadequate GWG, or underweight BMI. * *p* < 0.05, ** *p* < 0.001.

## Data Availability

The data presented in this study are available upon request from the corresponding author.
